# Protective Barriers Provided by the Epidermis

**DOI:** 10.3390/ijms24043145

**Published:** 2023-02-05

**Authors:** Sarah de Szalay, Philip W. Wertz

**Affiliations:** 1 Sarah de Szalay Consulting, LLC, Wesy Milford, NJ 07480, USA; 2 Dows Institute for Dental Research, College of Dentistry, University of Iowa, Iowa City, IA 52240, USA

**Keywords:** antimicrobial lipids, antimicrobial peptides, barrier function, epidermis, Langerhans cells, melanocytes

## Abstract

The skin is the largest organ of the body and consists of an epidermis, dermis and subcutaneous adipose tissue. The skin surface area is often stated to be about 1.8 to 2 m^2^ and represents our interface with the environment; however, when one considers that microorganisms live in the hair follicles and can enter sweat ducts, the area that interacts with this aspect of the environment becomes about 25–30 m^2^. Although all layers of the skin, including the adipose tissue, participate in antimicrobial defense, this review will focus mainly on the role of the antimicrobial factors in the epidermis and at the skin surface. The outermost layer of the epidermis, the stratum corneum, is physically tough and chemically inert which protects against numerous environmental stresses. It provides a permeability barrier which is attributable to lipids in the intercellular spaces between the corneocytes. In addition to the permeability barrier, there is an innate antimicrobial barrier at the skin surface which involves antimicrobial lipids, peptides and proteins. The skin surface has a low surface pH and is poor in certain nutrients, which limits the range of microorganisms that can survive there. Melanin and trans-urocanic acid provide protection from UV radiation, and Langerhans cells in the epidermis are poised to monitor the local environment and to trigger an immune response as needed. Each of these protective barriers will be discussed.

## 1. Permeability Barrier

The skin surface, including the linings and sweat gland ducts, provides anb extensive area for interactions between the skin and elements of its environment, including microorganisms [1]. The epidermis provides a number of protective mechanisms against physical and chemical assaults [2,3,4,5] and microbial colonization and invasion [6,7,8]. The permeability barroer provided by ceramides, cholesterol and free fatty acids in the intercellular spaces of the stratum corneum is the most fundamental of these protective mechanisms.

**Figure 1:**
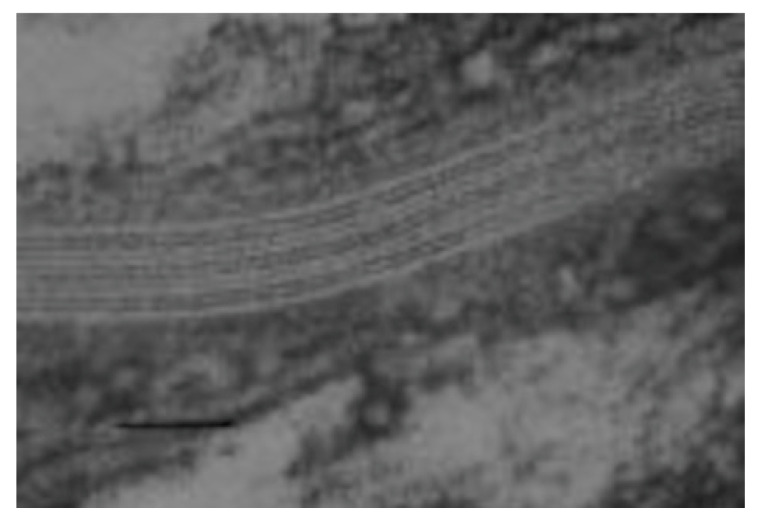
Transmission electron micrograph of an intercellular space in the stratum corneum [5]. The human skin specimen was fixed with ruthenium tetroxide. Bar = 50 nm.

Ceramides were first identified as epidermal components by Nicolaides in 1965 [9]. This was based on an infrared spectrum of a lipid fraction recovered from a thin-layer chromatogram and was added as a footnote to the proof of the manuscript. In the mid- to late 1970s, the group headed by G.M. Gray at Birmingham, UK, made significant progress toward elucidating the structures of the epidermal glucosylceramides from the viable portion of the epidermis and the ceramides from the stratum corneum (reviewed in [5]). This group showed that the epidermal ceramides are structurally heterogenous, and contain normal fatty acids ranging from 14- through 30-carbons in length with the 24- and 26-carbon entities being the most abundant. They also identified 24- and 26-carbon long α-hydroxyacids among the amide-linked fatty acids. The bases included sphingosines and dihydrosphingosines in the chain length range of 16 through 22 carbons. An 18-carbon phytosphingosine was also identified. These studies demonstrated that keratinocytes accumulate increasing amounts of lipid as they differentiate, and that the composition of the lipid alters with differentiation. The stratum corneum lipids consist mainly of ceramides, cholesterol and free fatty acids. Much of the accumulating lipid is packaged into small organelles called lamellar granules [10]. In the uppermost granular cells, the bounding membrane of the lamellar granules fuses into the plasma membrane and the granule contents are extruded into the intercellular space, where they form multilamellar arrays shown in Figure 1 [5]. It was clear from this work that glucosylceramides and sphingomyelin accumulated in the viable portion of the epidermis and were converted to ceramides late at the end of the differentiation process. Porcine epidermis was identified as a good model for human epidermis.

**Figure 2:**
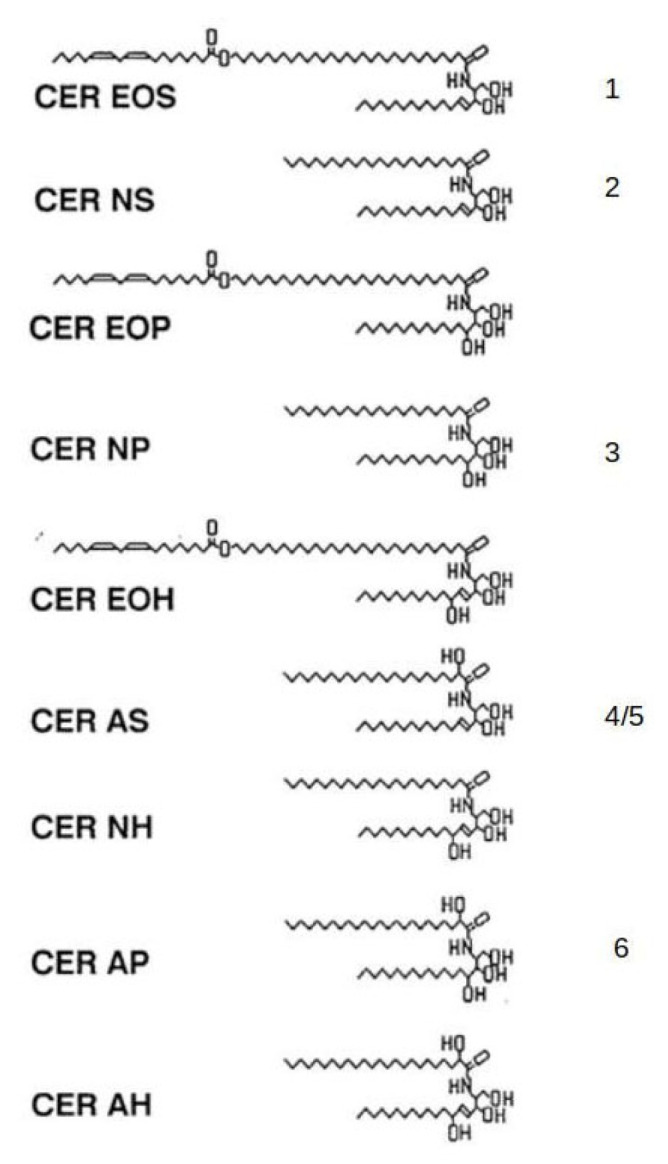
Ceramide structures from porcine and human stratum corneum [5]. The numbers (1–6) on the right indicate ceramides from porcine stratum corneum, while human ceramides are indicated on the left in the nomenclature of Motta et al. [12]. The porcine ceramide designated as 4/5 separates into two separate bands. The more mobile fraction contains mostly 20- through 28-carbon α-hydroxyacids amide-linked to sphingosine and dihydrosphingosine bases. The less mobile fraction is mostly α-hydroxy palmitic acid amide-linked to the same bases. In the human CER AS, there is an α-hydroxyacid-containing component with a more uniform chain length distribution.

In 1983, the first complete set of epidermal ceramide structures was published, and these structures are shown in Figure 2 [5]. The least polar and most unusual of these ceramides consisted of mainly 30- through 34-carbon ω-hydroxyacids amide-linked to a series of sphingosine and dihydrosphingosine bases with linoleic acid ester-linked to the ω-hydroxyl group [5]. X-ray diffraction studies of stratum corneum have revealed that the intercellular lipids are organized in a predominant repeat distance of approximately 13 nm [11]. This 13 nm repeat has been referred to as the long periodicity phase. Subsequent studies have demonstrated that the linoleate-containing acylceramide, or CER EOS in the nomenclature system of Motta et al., is required for formation of the long periodicity phase [11,12,13]. Electron diffraction has revealed that the lateral lipid packing is predominately orthorhombic [14].

The first complete set of human epidermal ceramides was reported in 2003, and these structures are shown in Figure 2 [5]. In contrast with the ceramides from pig, those from the human have three acylceramides. One of these is essentially the same as the one from pig, but one contains phytosphingosine as the base component and another contains 6-hydroxysphingosine as the base. In addition, the human ceramides include CER NH and CER AH, which are not found among the porcine ceramides. More recently, more detailed analyses of the stratum corneum lipids have been done by HPLC coupled with mass spectrometry [15].

**Figure 3:**
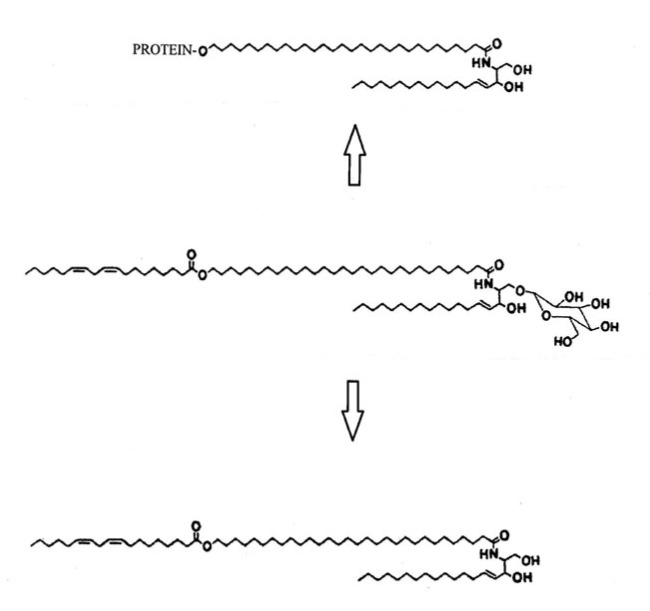
The linoleate-rich acylglucosylceramide (center) is the precursor of the acylceramide (bottom) and the covalently bound ω-hydroxyceramide [5]. This lipid is attached to the outer surface of the cornified envelope and has been referred to as the corneocyte lipid envelope, or CLE.

In the late 1980s, covalently bound ω-hydroxyceramides were reported in stratum corneum, and it was proposed that this lipid could be attached to the outer surface of the cornified envelope to form a lipid envelope [16]. Subsequent electron microscopic studies have supported this suggestion. This lipid is thought to be derived from the lamellar granule-associated linoleate-containing acylglucosylceramide shown in Figure 3 [5]. It has been suggested that the corneocyte lipid envelope may contribute to the chemical resistance of the stratum corneum and may also serve as a template upon which the lamellae formed by the free stratum corneum lipids orient themselves. It may have a role in cohesion of the stratum corneum, and it has been suggested that it may provide a semipermeable membrane around the corneocytes allowing passage of water but preventing the loss of other larger hygroscopic substances. It also may serve as a reservoir of the antimicrobial free long-chain bases under certain microbial stresses. The acylglucosylceramide is also the precursor of the acylceramide found in the intercellular spaces of the stratum corneum. The pathway by which the ω-hydroxyceramide becomes covalently attached to the cornified envelope has been reviewed [16].

Recently, a 1-O-acylceramide has been reported as a minor component of stratum corneum lipids [17]. The total of this lipid class is about 5% of the total acylceramide, which is 1% or less of the total stratum corneum lipid. These mainly consist of C18:1 sphingosine with amide-linked fatty acids ranging from 14- through 34-carbons in length, and fatty acids ranging from 14- through 30-carbons in length ester-linked to the primary hydroxyl group of the long-chain base [18]. Minor species contained C16:1, C17:1, C18:2 and C19:1 sphingosines. The functional role of this lipid family is not presently known.

An alternative mechanism by which the intercellular lamellae of the stratum corneum are formed has been proposed on the basis of cryo-transmission electron microscopic images [19,20]. In this alternative, there are no lamellar granules. There is membrane continuity from the endoplasmic reticulum through the intercellular lamellae. Five steps or stages with different lipid composition and organization have been identified in this membrane unfolding process. The cryoelectron microscopic images show a granular pattern reflecting the secretory apparatus of the uppermost granular cell. This makes a transition to a lamellar pattern with a 50–55 angstrom periodicity. This undergoes another transition to a lamellar pattern with a 20–25 angstrom periodicity between the first and second corneocytes at the bottom of the stratum corneum. Above this level of the stratum corneum, the periodicity of the lamellae is 110–120 angstroms.

## 2. Roles of Filaggrin

Filaggrin contributes to the permeability barrier, the water holding capacity of the stratum corneum and protection against UVB.

Profilaggrin is the highly phosphorylated, histidine-rich protein that constitutes the bulk of the keratohyalin granules [21]. It is also rich in arginine. It has a high molecular weight (greater than 220,000) and is highly phosphorylated. In the late stages of differentiation, profilaggrin is dephosphorylated and proteolytically converted into filaggrin (filament-aggregating protein) with a molecular weight of 37,000 [22]. Ten to twelve filaggrin molecules are produced from one profilaggrin molecule [22]. As the cornified envelope is forming, the positively charged filaggrin molecules bind to and aggregate the keratin filaments, causing flattening of the cell to form the corneocyte. After aggregation of the keratin filaments, the arginines in filaggrin undergo conversion to citruline through the action of peptidylarginine deiminases 1 and 3 [23]. This reduces the charge of the filaggrin molecule and allows it to unwind from the aggregated keratin, and it is proteolytically degraded to its component amino acids by a combination of bleomycin hydrolase, caspase −14 and calpain 1 [24,25]. The free glutamine released from filaggrin spontaneously cyclizes to form pyrrolidine carboxylic acid (PCA), and the histidine is converted to trans-urocanic acid by histidine ammonia-lyase [26]. PCA, urocanic acid and the other amino acids released by degradation of filaggrin are the major components of what has been called the natural moisturizing factor. The high concentration of small molecules within the corneocyte results in a high osmotic strength, which allows the corneocytes to effectively retain water. The lipid envelope on the outside of the cornified envelope and the intercellular lipid lamellae probably assist in holding water inside the corneocytes.

Mutations of the fillaggrin gene result in ichthyosis vulgaris and atopic dermatitis [27,28]. Decreased barrier function associated with these conditions can lead to contact allergies [29,30]. Atopic dermatitis often leads to asthma, food allergies and allergic rhinitis [31].

## 3. Antimicrobial Lipids and Lysosome

The pH of the skin surface of healthy adults is below 5 [8]. Several factors contribute to this acidification. One of these is the liberation of fatty acids from sebaceous triglycerides by microbial lipases in the follicles and on the skin surface [32,33]. The production of short chain fatty acids by some of the commensal microorganisms on the skin is a second mechanism for acidification [34,35,36]. The sodium–hydrogen exchanger 1 is another mechanism for lowering the pH of the stratum corneum [37]. Finally, production of lactic acid through anaerobic glycolysis as cells move outward from the basal layer will contribute to lowering the pH [38]. The acidic pH of the skin surface limits the range of microorganisms that can survive there because most microorganisms grow best at a more neutral pH.

The surface of the skin is relatively dry and nutrient-poor, which also limits the microorganisms that can survive there [39]. Some of the commensals that are part of the microbiome produce antimicrobial metabolites that protect against potential pathogens [34,39,40,41]. The skin microbiome interacts with the epidermis to influence the physical, chemical and innate immune barriers. These interactions have recently been reviewed [42,43].

The dermis contains sebaceous glands and sweat glands embedded in the reticular dermis, and both types of glands secrete their products onto the skin surface. The sebaceous glands and sweat glands secrete lipids and aqueous sweat onto the skin surface. Both of these secretions provide some antimicrobial components. Human sweat contains lysozyme, and apocrine sweat contains the antifungal lipid, undecylenic acid [44,45,46].

It has long been recognized that lipids at the skin surface have antimicrobial properties [47,48]. Burtenshaw demonstrated that ethyl ether extracts of the skin surface contained material that could kill *Staphylococcus aureus* and some other bacteria. He speculated that this might be attributed to free fatty acids and demonstrated that some free fatty acids and mixtures of fatty acids were antimicrobial. Several years later, the human sebaceous fatty acid fraction was shown to have antifungal activity against *Microsporum audouini*, the fungus that causes ringworm of the scalp [49]. Upon fractionation, the odd-carbon short chain fatty acids (C7:0, C9:0 and C11:0) were identified as the active components. This explained Rothman’s observation that young children who got ringworm of the scalp, cradle cap, often had recurrence of this infection until they reached puberty, which is associated with a major increase in sebum secretion. As noted previously, triglycerides are synthesized in the sebaceous glands, and fatty acids are released from these sebaceous triglycerides through the action of microbial lipases in the follicle and on the skin surface [34]. Subsequently, lauric acid (C12:0) and monolaurin were found to be uniquely antimicrobial against a range of Gram-positive bacteria [48]. The previous work of Rothman and associates demonstrated that lauric acid is among the fatty acids that are liberated from sebaceous triglycerides, and this was subsequently confirmed by gas–liquid chromatographic analysis [49,50]. Both lauric acid and sapienic acid (C16:1Δ6), one of the more abundant sebaceous fatty acids, were shown to be capable of killing Gram-positive bacteria as well as the Gram-negative gingival pathogens *Fusarium gingivalis* and *Porphyromonas gingivalis* [48]. Sebacous lipids are present in human saliva [48]. Under conditions where lauric acid produced nearly complete killing, other saturated fatty acids known to be present in human sebum (C8:0, C10:0, C14:0, C16:0, C18:0 and C20:0) were inactive or had some degree of bacteriostatic activity. Lauric acid also has activity against fungi and enveloped viruses [51,52]. Sapienic acid has not yet been tested for antiviral activity.

Monolaurin is available from numerous sources as a dietary supplement. One study sought the benefits of monolaurin use [53]. Three peer-reviewed cases in which human infections were successfully treated with monolaurin were found; however, these were all cases of topical infections. It was suggested that taking monolaurin orally may have no benefit other than the nutritional value. However, another study suggested that monolaurin may provide protection against COVID-19 [54]. In this study, 51 healthcare workers who were about to start working with COVID-19 patients were recruited. Blood samples were collected after enrollment. Within three weeks of working with COVID-19 patients, 24 of these subjects had contracted COVID-19. Metabolomic analysis identified 322 low molecular weight metabolites. Of these, 21 were elevated in the subjects who did not become infected relative to those who were infected. One of these molecules was monolaurin. A second molecule in this protective set was N,N-dimethylglycine. It was noted that these two molecules are available as nutritional supplements, which could potentially provide resistance to infection by SARS-CoV-2.

Half of the lipid molecules in the intercellular spaces are ceramides containing sphingosine, dihydrosphingosine, phytosphingosine or 6-hydroxysphingosine as the long-chain base component, and the cornified envelope of the corneocytes has a covalently attached ω-hydroxyceramide containing the same long-chain bases [16]. Ceramidase activity capable of hydrolyzing the amide linkage of ceramides to release free fatty acid and long-chain base have been identified in epidermis [7]. Free long-chain bases have been detected in human stratum corneum [55]. Free long-chain bases have been shown to be potent antimicrobial agents against Gram-positive bacteria, some Gram-negative bacteria and *Candida albicans* [56,57]. Several observations suggest that the covalently bound ω-hydroxyceramides in the corneocyte lipid envelope may serve as a source of free long-chain base. For one thing, as essential fatty acid deficiency developed in young pigs, the transepidermal water loss (TEWL) increased, and this increase in TEWL was accompanied by a decrease in the amount of covalently bound ω-hydroxyceramide with an increasing amount of covalently bound ω-hydroxyacid, suggesting ceramidase action [57]. In addition, in both atopic dermatitis and psoriasis, where barrier function is compromised, the proportion of covalently bound ω-hydroxyceramides is reduced and the proportion of covalently bound ω-hydroxyacids is elevated compared to normal control stratum corneum [16,57]. This may be a defense mechanism to provide increased antimicrobial protection in the face of a compromised barrier [16].

## 4. Antimicrobial Peptides

There are also antimicrobial peptides at the skin surface. The cathelicidin LL-37 is synthesized by keratinocytes and is the only known cathelicidin in human epidermis. The name, LL-37, is based on the fact that the sequence of 37 amino acids begins with two leucines at the amino terminal end [58]. At physiological pH, LL-37 has a net positive charge of +6. It has been shown by means of nuclear magnetic resonance that residues 2–31 form two helical regions with a bend at residue 15 [59]. It was shown that the entire amphipathic helical region associated with the negatively charged surface of micelles. This is in support of the mechanistic possibility that the LL-37 binds to the negatively charged membrane of a potential pathogen while the less structured carboxyl end of the molecule disrupts the integrity of the membrane. In normal human epidermis, the level of LL-37 is relatively low, but production is increased in response to inflammatory conditions or infection [60]. It has been shown that LL-37 is present in lamellar granules [61]. Lamellar granules, which deliver their contents to the intercellular space as described above, have also been shown to contain lysozyme [62]. LL-37 has broad antimicrobial activity against a variety of Gram-positive and Gram-negative bacteria, fungi and enveloped viruses [63].

There are four major antimicrobial β-defensins expressed in human epidermis: hBD1, hBD2, hBD3 and hBD4. The beta defensins are all cationic and contain six cysteine residues which form three disulfide linkages locking the peptide into a β-sheet structure [64]. Like cathelicidins, the defensins have broad antimicrobial activity against a range of Gram-positive bacteria, Gram-negative bacteria, fungi and some enveloped viruses [64,65]. HBD1 contains 36 amino acids and has a net charge of +4 at physiological pH [62]. The hDB2 peptide contains 36 amino acids and has a net charge of +6 [65]. Gram-negative bacteria and fungi are killed by hDB2 at low concentrations, but Gram-positive bacteria are much more resistant [66]. It has been shown that hBD2 is packaged into lamellar bodies [66]. This is likely the mechanism by which the cathelicidins and the β-defensins are delivered to the stratum corneum. Fatty acids liberated from sebaceous triglycerides by microbial lipases upregulate hBD2 [67]. The hDB3 peptide contains 44 amino acid residues and has a net charge of +11 [64]. Gram-positive and Gram-negative bacteria and fungi are effectively killed by hDB3. The hDB4 molecule contains 47 amino acids and has a net charge of +7 [62]. Some Gram-negative bacteria and some Gram-positive bacteria were killed with low doses of hBD4, while others were much more resistant. The hBD2, hBD3 and hBD-4 have all been experimentally upregulated when primary keratinocytes in culture were exposed to calcium to induce differentiation [68]. They were also upregulated by exposure to bacteria, interleukin-1 and TNF-alpha, or the phorbol ester, 12-O-tetradecanoyl-phorbol-13-acetate (TPA) [69]. TPA induces an inflammatory response. hBD1 is constituently expressed in human epidermis, but its expression can be increased by the presence of microbial molecules such as lipopolysaccharide. hBD2, hBD3 and hBD4 are expressed at low levels under unchallenged conditions, but expression can be induced by exposure to inflammatory conditions or microbial components including viruses. In addition to their direct antimicrobial action, hBD-1 can act as a chemokine to attract immune cells to the epidermis, thereby linking innate and active immunity [70]. As with cathelicidin LL-37, the defensins are thought to interact with negatively charged microbial or viral membranes by virtue of their positive charge.

A third category of antimicrobial peptides is a family of dermcidin-derived peptides. The dermcidin precursor is constitutively produced in eccrine sweat glands [68]. More recently, it has been reported that the dermcidin precursor is also synthesized by human sebocytes [71]. The dermcidin precursor contains 110 amino acids and is negatively charged [72]. The precursor protein is acted upon by cathepsin D in sweat to liberate two antimicrobial peptides from the carboxy terminal end [73]. DCD-1 contains 47 amino acids and DCD-1L contains an additional leucine. Both DCD-1 and DCD-1L have a net charge of −2. The peptides were highly active against *Escherischia coli*, *Escherischia faecalis*, *S.taphylococcus aureus* and *Candida albicans* [72]. DCD-1L was also shown to be active against *Acinetobacter baumannii*, including some antibiotic-resistant strains [74,75]. When sweat was collected from the skin surface, 14 additional DCD-derived peptides were identified [76]. The number of amino acids in these peptides ranged from 24 through 46, and the charge ranged from −2 to +1. This truncation is most likely mediated by proteases on the stratum corneum surface. The proposed mechanism of action requires zinc [77]. The monomeric DCD-1L molecules in sweat are unstructured. The positively charged amino terminus binds electrostatically to the negatively charged phospholipids of the microbial membrane and this interaction induces a transition from unstructured random coil to an alpha-helical conformation. Additional DCD-1L molecules aggregate to an oligomeric state which is stabilized by zinc. This aggregate forms a pore which results in cell death. DCD-1L has been shown to form a hexameric complex before insertion into the microbial membrane [77]. The orientation of this channel within the membrane is influenced by the membrane lipid composition [78,79]. In recent years, dermcidins have been implicated in the etiology of some cancers [80]. Seriniquinones, a class of drug derived from a marine bacterium, were found to be active against a number of melanoma cell lines [81]. The mechanism of action was inactivation of dermcidins.

Yet another antimicrobial protein synthesized constitutively and secreted by keratinocytes is the 14.5 kDa RNase 7 [82]. RNase 7 is effective in killing a range of Gram-positive bacteria, Gram-negative bacteria and yeast. Although it is constitutively expressed at a high level, higher expression can be induced by inflammatory mediators. RNase 7 is secreted by keratinocytes and accumulates at the skin surface. The antibacterial activity of RNase 7 is independent of the enzymatic activity and is localized to the amino terminal end of the molecule [83]. Peptides derived from the amino terminal end of human RNase 7 are highly active against *C. albicans*. The mechanism of action involves first binding to the cell wall followed by membrane permeabilization [84]. RNase 7 can bind self-DNA, which could be released from damaged keratinocytes, and activate keratinocytes to produce and secrete the chemokine, IP-10. IP-10 attracts a range of immune cells to the epidermis, and this enhances the antiviral defense [83].

The S100 antimicrobial proteins are so named because they are soluble in 100% saturated aqueous ammonium sulfate at neutral pH [85]. There are currently 25 known S100 proteins expressed among vertebrates [86]. These proteins are generally small (10–12 KDa). They have two looped calcium binding sites, each of which is flanked by alpha-helical domains. There is a hinge domain between the central two alpha-helical domains, and there is a high degree of amino acid sequence homology from the amino terminal end through the alpha-helical domain closest to the carboxyl terminal end. The amino acid sequence near the amino terminus is variable. The active forms exist as antiparallel dimers, which are most often homodimers. Three of these S100 proteins (psoriasin, calmodulin and koebnerisin) are expressed in human keratinocytes. The S100 proteins play numerous roles in the immune system [87]. It has been suggested that certain S100 proteins collected by nasal swabbing could be useful as a prognostic marker of COVID-19 infection.

Psoriasin (S100A7) is expressed at low levels in normal human keratinocytes, but its expression is much upregulated in psoriatic epidermis and in atopic dermatitis [88,89]. Psoriasin is most effective in killing *E. coli*, but at higher concentrations it can kill other bacteria. At pH below 6, it killed *E. coli* and *Bacillus megaterium* by membrane permeabilizing and pore formation; however, at neutral pH it killed *E. coli* without permeabilization. In psoriatic subjects, the level of psoriasin in serum was significantly elevated relative to that in control serum; however, the degree of elevation did not correlate with disease severity [90]. In several superficial fungal infections, it has been shown that psoriasin is elevated [91,92,93]. Although direct activity against fungus was not demonstrated, it was found that psoriasin binds to β-glucan. This is a component of the *C. albicans* cell wall, and psoriasin thereby prevents *C. albicans* from adhering to the host surface. Subjects with anorexia nervosa do not suffer skin infections, which are common in generally malnourished individuals. This unusual disease resistance may be attributed, at least in part, to upregulation of both psoriasin and RNase 7 [94,95].

The active form of calprotectin is a heterodimer (S100A8/A9) [65]. Calprotectin, as well as the other S100 proteins, is a calcium-binding protein; however, it can also bind other divalent metal cations including Mn^+2^, Cu^+2^, Fe^+2^, Ni^+2^ and Zn^+2^ [96]. In skin wounding or infection, calprotectin provides what is called nutritional immunity by sequestering these essential nutrients [97]. Calprotectin has been shown to be effective in killing *S. aureus*, *S. epidermidis*, *E. coli* and *C. albicans* in vitro [97]. The C-terminal end of the one calprotectin chain has an amino acid sequence identical to the N-terminal end of neutrophil immobilizing factors. This may be significant for attracting granulocytes [96]. The severity of psoriasis is subjectively evaluated by the Psoriasis Area and Severity Index. The level of calprotectin at the psoriatic skin surface has been shown to correlate strongly with this index.

Koebnerisin (S100A15) has an amino acid sequence very similar to that of psoriasin [63]. It probably contributes to nutritional immunity in a manner similar to calprotectin. Koebnerisin is overexpressed in several inflammatory skin conditions including psoriasis, rosacea, hidradenitis suppurativa (acne inversa) and acne vulgaris [65,97,98].

SPINK9 is an inhibitor of kallikrein-like proteases and is involved in the regulation of desquamation. It is upregulated in type 1 diabetes [99]. Six N-terminal variants of SPIMK9 have been identified in human stratum corneum [100]. Three of these variants kill various strains of *E. coli*, but not yeast or other bacteria.

Increasing development of antibiotic-resistant microorganisms creates a need to identify new antimicrobials and, in this regard, antimicrobial peptides have gotten the attention of the pharmaceutical and agricultural industries [101,102,103,104,105]. More than 3000 naturally occurring antimicrobial peptides have been identified, and there is a database containing 24,243 natural and synthetic antimicrobial peptides [106]. More than 60 antimicrobial peptides have been approved by the FDA [107]. The natural antimicrobial peptides are susceptible to proteolytic degradation and are hemolytic [108,109]. The synthetic antimicrobial peptides are based on natural antimicrobial peptides in attempts to minimize toxicity, improve stability and retain or enhance activity [108,109,110,111].

## 5. Langerhans Cells

In human epidermis, the link between innate immunity and adaptive immunity is provided largely by the Langerhans cells [112,113]. Although they can be found throughout most of the viable epidermis, Langerhans cells are most abundant in the spinous layer [114]. Langerhans cells are dendritic, and the dendrites contact nearby keratinocytes. The extension and retraction of the dendrites is a dynamic process called dendrite surveillance extension and retraction cycling [115,116]. This process allows Langerhans cells to survey the environment throughout the viable epidermis and, by penetrating through the tight junctions between granular cells, to reach the stratum corneum [110,114,115,116]. Langerhans cells are equipped with various pattern recognition receptors including toll-like receptors (TLR2, TLR4 and TLR5) and the mannose-specific lectin langerin [110,111]. The toll-like receptors provide for bacterial recognition, while langerin recognizes certain viruses. Under normal conditions, Langerhans cells collect endogenous antigens and antigens of the microbiome and migrate to skin-draining lymph nodes [112]. Under these conditions, presentation of the antigens to naive T cells results in tolerance. The population of Langerhans cells in the epidermis under nonstressed conditions is maintained by in situ replication of Langerhans cells in the epidermis [112]. In cases of infection, the naive T cells in the lymph nodes are activated to produce antibodies against pathogens. During inflammation, some of the Langerhans cells that replace those migrating to lymph nodes arise from monocytes from the circulation [111]. Whether antigen presentation results in immune response or tolerance is dependent upon the state of maturity of the Langerhans cell [113]. In aged human skin, among other things, the number of Langerhans cells in the epidermis is decreased, and the incidence of skin infections increases [117,118].

## 6. Melanocytes

Melanocytes are dendritic cells found in the basal layer of the interfollicular epidermis [118]. They contain lysosome-related organelles called melanosomes which are the sites of pigment synthesis [119,120,121]. Melanin synthesis begins by the oxidation of tyrosine to DOPA and the oxidation of DOPA to DOPAquinone. These two oxidations are catalyzed by tyrosinase or either of two paralogues (Trp1 and Trp2) to produce the pigment eumelanin [120,121]. Tyrosinase is a copper-requiring enzyme [119]. Trp 1 is thought to also be a copper-requiring enzyme, while Trp 2 requires zinc. These tyrosinase paralogues determine the type and amount of melanin produced. A series of spontaneous reactions leads from DOPAquinone to the brown-black pigment eumelanin. Alternatively, DOPAquinone can react with cysteine to form cysteinylDOPA, which leads to formation of the yellow-red pigment pheomelanin. Numerous endogenous molecules can influence the activity of melanosomes [121]. Melatonin inhibits melanin formation by lowering tyrosinase activity levels [121]. The melanosomes travel through the dendrites and are transferred into the keratinocytes. Several mechanisms have been proposed for the actual transfer from the melanocyte dendrite into the cytoplasm of the keratinocyte [122,123]. Melanin/pheomelanin provides a degree of protection against ultraviolet-induced damage. Consistent with the role of protecting against UV-induced DNA damage, the melanosomes tend to cluster into a cap above the nucleus [123].

In addition to protective pigmentation, trans-urocanic acid produced during degradation of filaggrin is an ultraviolet chromophore [124]. When it absorbs UV, it isomerizes to cis-urocanic acid.

## Data Availability

Not applicable.
